# Continuous measurement of interstitial glycaemia in professional female UCI world tour cyclists undertaking a 9‐day cycle training camp

**DOI:** 10.1002/ejsc.12201

**Published:** 2024-09-28

**Authors:** Ross Hamilton, Olivia M. McCarthy, Stephen C. Bain, Richard M. Bracken

**Affiliations:** ^1^ Applied Sport, Technology, Exercise and Medicine Research Centre Swansea University Swansea UK; ^2^ Steno Diabetes Centre Copenhagen Copenhagen University Hospital Herlev Denmark; ^3^ Medical School Swansea University Swansea UK; ^4^ Faculty of Science and Engineering Health Technology and Solutions Interdisciplinary Research Institute Swansea University Swansea UK

**Keywords:** continuous glucose monitoring, endurance exercise, female athletes, glycaemia, professional cyclists

## Abstract

Nine cyclists (age: 26 ± 5 years, height: 168 ± 5 cm and mass 58.5 ± 4.5 kg) were observed using continuous glucose monitoring devices throughout a training camp. Interstitial glucose [iG] data were captured via the Abbott libre sense biosensor (Abbott Laboratories) and paired with the Supersapiens software (TT1 Products Inc.). [iG] data were split into time ranges, that is, overall (24‐hourly), day‐time (06:00–23:59), night‐time (00:00–05:59) and exercise. [iG] data were stratified into percentage of time, below range ([TBR] < 70 mg/dl), in range ([TIR] 70–140 mg/dl) and above range ([TAR] ≥ 141 mg/dl). Differences in diurnal and nocturnal data were analysed via repeated measures analysis of variance and paired *t*‐tests where appropriate. *p*‐value of ≤0.05 was accepted as significant. Riders spent an average of 3 ± 1% TAR, 93 ± 2% TIR and 8 ± 3% TBR. Mean 24 h [iG] was 93 ± 2 mg/dl with a coefficient of variation (CV) of 18 ± 1%. Mean (day: 95 ± 3 vs. night: 86 ± 3 mg/dl and *p* < 0.001) and CV (day: 18 ± 1 vs. night: 9 ± 1% and *p* < 0.001) in [iG] were higher during the day‐time hours. TAR was greater during the day (day: 3 ± 1 vs. night: 0 ± 0% and *p* < 0.001) but TBR and TIR were similar. Glucose levels below the clinical range may have implications for those without diabetes and warrants further investigation.

## Highlights

- The data provided in this study offer novel insight into the daily glycaemic responses of female cyclists engaged in consecutive days' worth of heavy exercise training as part of a performance camp.
- % Time above range was greatest during exercise whereas, the % time below range was at its lowest. This may offer some insight into the demands of exercise and glucose variability.
- Riders maintained a high percentage of time spent in range, yet displayed some time <70 mg/dl, that is, hypoglycaemia. A proportionate amount of time was spent with glucose levels below the clinical range, the potential health and/or sports performance implications of this in athletic individuals without diabetes warrants further investigation.

## 1 INTRODUCTION

Historically, scientific understanding of the demands of professional cycling has been obtained from predominately male riders with a clear under‐representation of female cyclists in research and practice. Encouragingly, there has been an increase in the number of professional female cycling teams now competing in previously male‐only events (Sanders et al., 2018; The Cyclists' Alliance, 2022).

In the limited number of exercise science studies that have explored sex comparisons, many are performed in the controlled setting of the exercise laboratory (Clavel et al., 2022; Doering et al., 2019; Hawley et al., 1994; Herrington et al., 2012; Thomas et al., 2017). While offering a greater degree of control, laboratory‐based protocols and environmental conditions often fail to reflect the real‐world scenarios that are habitually undertaken in the field and suffer from poor ecological validity. Thus, observations of cyclists in their habitual training or race environments are important in shedding new light on the demands of the female athlete.

Stores of carbohydrate and circulating glucose are the preferred fuels during intense exercise and it is well recognised that maintaining adequate supply is essential for optimising performance (Coggan & Coyle, 1987, 1989; Jeukendrup, 2014; Jeukendrup & Jentjens, 2000; Jeukendrup, Raben, et al., 1999; Jeukendrup, Wagenmakers, et al., 1999). The relatively recent development of continuous glucose monitoring (CGM) provides real‐time feedback of interstitial glucose [iG] concentrations via subcutaneous sensors viewed by a mobile phone application or reader. Though originally developed for the therapeutic management of people with diabetes, these technologies have recently grown in popularity as a ‘biofeedback tool’ in athletes (Bowler et al., 2022; Holzer et al., 2022). While studies have noted the potential utility of CGM in an exercising context, there is currently very little evidence of its utility in an applied sporting setting (Holzer et al., 2022; Kinrade & Galloway, 2021; Klonoff et al., 2022; Podlogar & Wallis, 2022). Although the use of CGM is prohibited in racing by the Union Cycliste Internationale (UCI), gathering information under training conditions might be valuable in progressing our understanding of the glycaemic demands of sports performance in elite level athletes. The potential to amalgamate data from physical activity wearables, for example, mobile power meters and heart rate monitors presents opportunities to explore how glycaemia might be influenced by endurance exercise stress in competitive athletes.

With this in mind and given the inherent sex differences in physiological and metabolic responses to exercise (Cano et al., 2022; Elliott‐Sale et al., 2021; Tarnopolsky et al., 1990; Wismann & Willoughby, 2006), more female‐focused research in professional cycling is warranted in an attempt to bridge current knowledge gaps.

**Aim**: To characterise the glycaemic variability of professional female UCI world tour cyclists using continuous iG monitoring over a 9‐day cycle training camp.

## 2 METHODS

### 2.1 Study design

This was an observational, exploratory study involving nine professional female UCI tour riders. Ethical approval was granted by the Swansea University research ethics committee. The study was carried out in accordance with the Declaration of Helsinki and International Conference on Harmonisation and Good Clinical Practice. Only data collected from cyclists that provided informed consent were included in the study.

### 2.2 Cycle training regimen

Data were collected over a 9‐day training camp undertaken in January in Majorca, Spain. Throughout this period, riders performed daily exercise training sessions that were individualised and prescribed by the team's sports performance coaches. Some riders completed supplemental sessions aimed at addressing injury rehabilitation. The group took one rest day (day 4) and one reduced riding day (day 7), the latter of which was a sponsor‐related online cycling event. A summary of the grouped mean training loads throughout the week is detailed in Table 1.

**TABLE 1:** Summary exercise data for each day of the training camp (*n* = 9 riders).

Day	Duration (h:min:s)	Distance (km)	Heart rate (bpm)	Power (watts)	Speed (km/h)
1	03:47:25 ± 0:03:36	115.5 ± 0.4	144 ± 8	159 ± 19	32.0 ± 0.4
2	03:03:57 ± 1:14:59	109.4 ± 9.9	143 ± 7	164 ± 16	33.7 ± 1.3
3	05:46:48 ± 0:09:49	158.6 ± 12.3	134 ± 9	147 ± 19	28.9 ± 1.9
4	Rest				
5	04:11:38 ± 0:21:11	104.1 ± 7.1	140 ± 5	156 ± 14	26.5 ± 1.11
6	05:12:04 ± 0:23:42	144.4 ± 13.4	138 ± 9	164 ± 14	29.8 ± 0.34
7	00:40:37 ± 0:05:46	12.7 ± 7.8	137 ± 16	134 ± 4	19.3 ± 9.29
8	04:04:53 ± 0:26:42	111.0 ± 10.1	138 ± 8	144 ± 27	28.8 ± 0.86
9	06:21:56 ± 1:33:09	173.2 ± 38.1	133 ± 5	147 ± 29	28.5 ± 5.66
**Mean ± SD**	**04:15:03 ± 1:43:50**	**120.3 ± 43.8**	**138 ± 9**	**153 ± 22**	**29.0 ± 4.6**

*Note*: Data are reported as mean ± SD.

The riders were on a training camp in the early stage of the season focussing mostly on the low‐intensity training volume. This was the first group meet‐up of the year and, for several riders, their first involvement with the team. All training data were collected via individual rider head units and power meters. Each rider's data was imported to the Training Peaks application (Training Peaks, Peaksware LLC), then downloaded and sent to the research team for retrospective analyses. For each session, the head units recorded distance (km), speed (km/h), power (watts) and heart rate (beats per minute).

### 2.3 Computation of glycaemic data

All iG data were recorded via the Abbott libre sense biosensor CGM (Abbott Laboratories). The sensor was applied to the subcutaneous fat pad located over the triceps brachii as per manufacturer instructions. The CGM device was paired to the Supersapiens software application (TT1 Products Inc.), which was installed on the participant's smart phone. Raw CGM data were exported and analysed via Excel 2019 (Microsoft Corp.). Daily [iG] data were retrospectively split into distinct time ranges, that is, overall (24‐hourly), day‐time (06:00–23:59), night‐time (00:00–05:59) and exercise; defined as the data points that fell within the in‐ride time‐frame provided from each rider's head unit.

Group means were calculated for [iG] concentrations (mg/dl) and indices of glycaemic variability, that is, the coefficient of variation (CV) and standard deviation (SD). [iG] data were also stratified into a percentage of time spent in specific glycaemic ranges pre‐defined by the Supersapiens application: time below range ([TBR] < 70 mg/dl), time in range ([TIR] 70–140 mg/dl) and time above range ([TAR] ≥ 141 mg/dl).

### 2.4 Statistical analyses

All statistical analyses were carried out using the SPSS V 28.0 statistical software and Graphpad Prism V 9.5. All data were checked for normality. Data are presented as mean ± SD. Differences between variables across the days of the camp were assessed using a repeated measures one‐way analysis of variance (ANOVA). A two‐way ANOVA was used to discern differences between day‐ and night‐time variables as the camp duration progressed. Pearson's product moment correlation of coefficient test was used to explore relationships between exercise variables. A *p*‐value of ≤0.05 was accepted as a statistically significant difference or relationship.

## 3 RESULTS

### 3.1 Participants

Nine female UCI World tour riders (age: 26 ± 5 years, height: 168 ± 5 cm and mass: 58.5 ± 4.5 kg) took part in the study.

### 3.2 Exercise training data

Grouped mean daily exercise duration was 4:15:03 ± 1:43:50 h during the camp. Riders completed a mean of 7 ± 2 rides over the 9‐day period. During camp, riders had one complete rest day (day 4) and 1 day with a short session of active recovery (day 7). The mean distance covered per training session was 116.11 ± 48.95 km. Mean heart rate during exercise was 138 ± 4 bpm. Mean power output was 152 ± 10 W. Summary exercise training data are displayed in Table 1.

### 3.3 24‐h glucose

The group mean [iG] for the nine riders across the 9‐day period was 93 ± 2 mg/dl, with SD of 17 ± 1 mg/dl and CV of 18 ± 1%. There were no [iG] differences between days (all *p* ≥ 0.05 and Table 2). Maximum [iG] value was 158 ± 7 mg/dl and occurred during the day‐ rather than night‐time hours. Minimum value was 61 ± 2 mg/dl. Each 24‐h average [iG] was similar across 9 days (*p* = 0.164 and Table 2).

**TABLE 2:** Summary of group mean glycaemic parameters from all nine riders throughout the entire 9‐day training camp when data have been treated as an overall 24‐h period, a day‐time period (06:00–23:59), a night‐time period (00:00–05:59) and as an exercise period (in‐ride data based on the duration of each individual cycling session).

Glycaemic parameter	Day 1	Day 2	Day 3	Day 4	Day 5	Day 6	Day 7	Day 8	Day 9	*p*‐value
Overall (24 h)										
Max (mg/dl)	164 ± 9	161 ± 20	156 ± 27	145 ± 20	150 ± 17	157 ± 27	154 ± 32	161 ± 24	169 ± 24	*p* = 0.199
Mean (mg/dl)	96 ± 9	93 ± 9	91 ± 8	90 ± 10	93 ± 11	92 ± 11	91 ± 12	92 ± 11	96 ± 7	*p* = 0.165
Min (mg/dl)	61 ± 8	63 ± 8	58 ± 5	60 ± 8	61 ± 8	63 ± 7	64 ± 8	59 ± 6	61 ± 3	*p* = 0.289
SD (mg/dl)	18 ± 3	16 ± 3	18 ± 4	15 ± 4	15 ± 3	16 ± 3	16 ± 6	18 ± 4	19 ± 5	*p* = 0.168
CV (%)	19 ± 2	17 ± 3	20 ± 4	17 ± 4	16 ± 3	17 ± 2	18 ± 5	19 ± 4	20 ± 5	*p* = 0.211
TAR (%)	3 ± 3	3 ± 3	3 ± 3	2 ± 2	2 ± 2	3 ± 3	4 ± 4	4 ± 5	3 ± 3	*p* = 0.345
TIR (%)	89 ± 6	96 ± 2	92 ± 6	92 ± 10	95 ± 5	95 ± 5	93 ± 8	93 ± 3	95 ± 3	*p* = 0.192
TBR (%)	6 ± 6	7 ± 14	11 ± 13	12 ± 18	8 ± 12	8 ± 13	10 ± 19	9 ± 15	3 ± 3	*p* = 0.302
Day‐time (06:00–11:59)										
Max (mg/dl)	164 ± 9	161 ± 20	156 ± 27	145 ± 20	150 ± 17	157 ± 27	154 ± 32	161 ± 24	169 ± 24	*p* = 0.144
Mean (mg/dl)	99 ± 10	96 ± 9	93 ± 10	93 ± 12	94 ± 11	94 ± 12	95 ± 14	95 ± 11	100 ± 8	*p* = 0.144
Min (mg/dl)	62 ± 8	63 ± 8	58 ± 5	61 ± 9	61 ± 8	63 ± 7	65 ± 8	60 ± 6	61 ± 3	*p* = 0.318
SD (mg/dl)	19 ± 3	17 ± 3	17 ± 4	16 ± 3	16 ± 3	17 ± 3	17 ± 6	18 ± 5	20 ± 6	*p* = 0.188
CV (%)	19 ± 2	18 ± 3	20 ± 4	17 ± 4	17 ± 3	18 ± 3	18 ± 5	20 ± 4	20 ± 6	*p* = 0.222
TAR (%)	4 ± 5	3 ± 3	3 ± 4	1 ± 2	2 ± 2	3 ± 4	3 ± 5	2 ± 3	3 ± 4	*p* = 0.429
TIR (%)	90 ± 6	93 ± 5	88 ± 10	88 ± 15	91 ± 10	90 ± 11	76 ± 3	90 ± 10	91 ± 7	*p* = 0.367
TBR (%)	6 ± 6	4 ± 6	9 ± 10	11 ± 15	7 ± 11	7 ± 12	10 ± 11	8 ± 11	6 ± 8	*p* = 0.172
Night‐time (00:00–05:59)										
Max (mg/dl)	106 ± 10	104 ± 13	114 ± 16.	106 ± 19	111 ± 22	104 ± 11	99 ± 13	107 ± 17	120 ± 21	*p* = 0.345
Mean (mg/dl)	86 ± 9	86 ± 10	87 ± 13	84 ± 12	92 ± 17	84 ± 9	82 ± 9	84 ± 11	87 ± 6	*p* = 0.200
Min (mg/dl)	66 ± 11	69 ± 12	68 ± 9	69 ± 11	80 ± 23	70 ± 8	70 ± 9	67 ± 11	74 ± 3	*p* = 0.279
SD (mg/dl)	7 ± 2	7 ± 2	9 ± 4	8 ± 4	8 ± 5	69 ± 2	6 ± 2	8 ± 5	9 ± 4	*p* = 0.364
CV (%)	8 ± 3	8 ± 3	11 ± 4	10 ± 5	10 ± 5	9 ± 4	8 ± 4	8 ± 4	10 ± 4	*p* = 0.544
TAR (%)	0 ± 0	0 ± 0	0 ± 0	0 ± 0	1 ± 3	0 ± 0	0 ± 0	0 ± 0	0 ± 0	*p* = 0.408
TIR (%)	91 ± 17	91 ± 23	86 ± 28	85 ± 28	92 ± 16	91 ± 19	89 ± 25	87 ± 30	100 ± 1	*p* = 0.989
TBR (%)	9 ± 17	9 ± 23	13 ± 28	15 ± 28	6 ± 16	9 ± 19	11 ± 25	13 ± 30	0 ± 1	*p* = 0.535
Exercise										
Max (mg/dl)	162 ± 18	149 ± 29	123 ± 17	Rest	138 ± 19	159 ± 30	127 ± 14	145 ± 35	152 ± 31	*p* = 0.304
Mean (mg/dl)	125 ± 12	109 ± 16	93 ± 11	Rest	107 ± 17	104 ± 16	104 ± 16	109 ± 23	103 ± 14	*p* = 0.234
Min (mg/dl)	93 ± 14	73 ± 10	63 ± 8	Rest	75 ± 17	74 ± 15	81 ± 9^a^	72 ± 14	61 ± 2^a^	*p* = 0.022
SD (mg/dl)	15 ± 3	16 ± 7	12 ± 3	Rest	11 ± 2	14 ± 3	14 ± 2	15 ± 8	15 ± 4	*p* = 0.690
CV (%)	12 ± 3	15 ± 5	13 ± 3	Rest	11 ± 1	14 ± 2	14 ± 0	13 ± 5	14 ± 2	*p* = 0.403
TAR (%)	21 ± 15	8 ± 9	0 ± 1	Rest	5 ± 10	6 ± 10	0 ± 0	15 ± 20	3 ± 4	*p* = 0.120
TIR (%)	79 ± 15	89 ± 8	93 ± 8	Rest	94 ± 9	92 ± 9	100 ± 0	84 ± 20	94 ± 4	*p* = 0.281
TBR (%)	0 ± 0	2 ± 5	7 ± 9	Rest	4 ± 9	3 ± 4	0 ± 0	3 ± 4	2 ± 4	*p* = 0.698

*Note*: TAR: the percentage of time spent with interstitial glucose levels above the target range (≥141 mg/dl). TIR: the percentage of time spent with interstitial glucose levels within the target range (70–140 mg/dl). TBR: the percentage of time spent with interstitial glucose levels below the target range (<70 mg/dl). Data are displayed as mean ± SD.

Abbreviations: CV, coefficient of variation; iG, interstitial glucose; Max, maximum; Min, minimum; SD, standard deviation.

^a^ Indicates a statistical difference between days for the respective glycaemic parameter (*p* ≤ 0.05).

**FIGURE 1:**
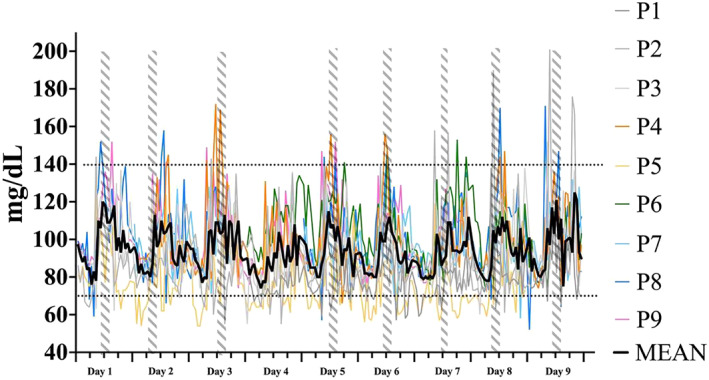
Grouped mean [iG] responses across each of the 9 days of training camp. Data are displayed as the mean (black line) and individual traces (coloured lines) in daily [iG] concentrations across each day of camp. Minor ticks on the *X*‐axis indicate 6 h (quarterly) time periods. The timing of each exercise session is indicated via the vertical dashed lines. Day 4 was a rest day whereas, day 7 had some short activity. The euglycaemic range (70–140 mg/dl) is indicated by the two parallel dashed lines running horizontally across the graph. iG, interstitial glucose.

Figure 1 displays grouped mean [iG] concentrations across camp whereas, Table 2 provides information on each glycaemic parameter over a 24‐h period on a day‐by‐day basis.

### 3.4 Day‐time glucose

Mean [iG] during day‐time hours was 95 ± 3 mg/dl, with mean SD of 18 ± 1 mg/dl and mean CV 18 ± 1%. Maximum [iG] value was 157 ± 7 mg/dl and minimum value was 61 ± 2 mg/dl. There were no significant differences in any [iG] metric between each 24‐h period (Table 2).

### 3.5 Night‐time glucose

The mean [iG] during the night‐time period was 86 ± 3 mg/dl, with a SD of 8 ± 1 mg/dl and CV of 9 ± 1%. Mean maximum night‐time [iG] value was 108 ± 6 mg/dl whereas, the mean minimum value during the night was 70 ± 4 mg/dl. There were no significant differences in any [iG] metric between each 6 h night‐time period (Table 2).

### 3.6 Exercise glucose

The mean [iG] during exercise was 108 ± 9 mg/dl with a SD of 14 ± 2 mg/dl and CV of 13 ± 2%. The mean maximum in‐ride [iG] value was 144 ± 14 mg/dl while the mean minimum value was 74 ± 10 mg/dl. There were no significant differences in any [iG] metric between each exercise period (Table 2).

### 3.7 Day‐time versus night‐time glucose comparisons

When comparing day‐time versus night‐time periods, a significant main effect was detected as the camp progressed (*p* = 0.037). Mean [iG] was higher during the day‐time hours (day: 95 ± 3 vs. night: 86 ± 3 mg/dl and *p* < 0.001). The SD (day: 18 ± 1 vs. night: 8 ± 1 mg/dl and *p* < 0.001) and CV (day: 18 ± 1 vs. night: 9 ± 1% and *p* < 0.001) of [iG] were both higher during the day‐time hours as was the TAR (day: 3 ± 1 vs. night: 0 ± 0% and *p* < 0.001). Both the TBR (day: 8 ± 3 vs. night: 10 ± 5% and *p* = 0.165) and TIR (day: 89 ± 5 vs. night: 90 ± 5% and *p* = 0.364) were similar between the day‐ and night‐time periods.

### 3.8 Relationships between glycaemia and exercise performance metrics

Table 3 details relationships between exercise glycaemic variables with the exercise performance metric. No associations were observed between in‐ride [iG] variables and exercise performance metrics on the same day. When observing the relationship between glycaemic variables from the preceding night‐time period to the subsequent day's exercise, there were no significant associations. There were also no associations found between exercise performance metrics each day and the [iG] metrics observed during the subsequent night‐time periods.

**TABLE 3:** Relationships between glycaemic variables and exercise performance metrics.

	[iG] Mean	TAR	TIR	TBR
Same day [iG]				
Power	0.19	0.18	−0.10	−0.23
HR	0.04	−0.11	0.25	−0.21
Duration	−0.12	−0.11	0.04	0.13
Night–day [iG]				
Power	0.03	0.10	−0.01	−0.01
HR	0.23	−0.03	0.06	−0.06
Duration	0.12	0.10	0.002	−0.01
Day–night [iG]				
Power	0.08	0.15	0.11	−0.12
HR	0.28	0.01	0.19	−0.20
Duration	0.12	0.04	0.08	0.08

*Note*: Same day: in ride [iG] and in ride exercise performance metrics. Night to day: Preceding night [iG] with subsequent days' exercise performance metrics. Day–night: daytime exercise performance metrics with subsequent nights' [iG]. HR: heart rate. TBR: the percentage of time spent with interstitial glucose levels below the target range (<70 mg/dl). TIR: the percentage of time spent with interstitial glucose levels within the target range (70–140 mg/dl). TAR: the percentage of time spent with interstitial glucose levels above the target range (≥141 mg/dl).

## 4 DISCUSSION

This study sought to characterise the glycaemic demands of an intensive training camp in professional female UCI world tour riders using CGM devices. These data provide a novel insight into the daily glycaemic responses of female cyclists engaged in consecutive days' worth of heavy exercise training as part of a performance camp.

Overall, riders in the present study spent a proportionately high percentage of their time (93 ± 2%) with [iG] levels in the ‘clinically defined’ target range, that is, 70–140 mg/dl with average euglycaemic values of 93 ± 2 mg/dl and little evidence of pronounced glycaemic variability (CV ∼ 18%). This carried over into the exercise period, with a mean iG concentration of 108 ± 93 mg/dl and a CV of ∼13%. Similar findings of tight glycaemic control during exercise have been observed in studies investigating mixed‐sex ultrarunners competing in single‐stage events (Hargreaves et al., 1984; Ishihara et al., 2020; Kulawiec et al., 2021; Sengoku et al., 2015). For example, Ishihara et al. (2020) noted normoglycemic iG concentrations in their cohort of runners throughout a 160 km ultramarathon event using an intermittent CGM device (All runners [*n* = 10]: 134 ± 19 mg/dl with a CV of 14.0%. Female only runners [*n* = 3]: 124 ± 18 mg/dl with a CV of 14.2%). Kinrade and Galloway (2021) also observed mean euglycaemic [iG] levels in mixed‐sex ultra‐endurance runners (*n* = 14) undertaking a continuous 24 h event (i.e., 124 ± 1 mg/dl). Important caveats that prevent direct inter‐study comparisons include differences in the use of CGM devices, glycaemic thresholds, time capture periods and exercise disciplines. Nevertheless, collectively they provide insight as to the seemingly tight level of glycaemic control that can be maintained under metabolically challenging circumstances. During exercise, both endogenous (glycogenolysis and gluconeogenesis) (Jeukendrup, Raben, et al., 1999; Kjaer et al., 1984) and exogenous (dietary carbohydrate intake) (Jeukendrup et al., 2006; Sengoku et al., 2015) inputs contribute significantly to the maintenance of glucose homoeostasis at a time when skeletal muscle tissue fuel demands are increased exponentially. The exploratory, observational nature of this study precluded access to information around endogenous and exogenous fuel use during exercise. However, the integration of continuous CGM over consecutive days' worth of data capture including daily bouts of cycle training expands our current knowledge base of glycaemia in an all‐female elite cycling cohort.

This study focussed on collecting measures of glycaemic variability throughout a 9‐day training camp. While others have also tracked some measures of variability (Francois et al., 2018; Thomas et al., 2016), few have collected data during the recovery period post‐exercise in a free living, real‐life training camp environment. CGM allows for a constant stream of data, which improves the ability to detect rapid fluctuations which might be missed if adopting a fixed timepoint collection schedule, which would be typical of finger prick sampling, the impracticability of which makes for difficulty in obtaining real‐time information.

A difference in variability was identified between the day‐ and night‐time periods. Both SD and CV were significantly different as well as the TAR. While [iG] was elevated during exercise, the maximum and minimum concentrations were experienced outside of exercise but within the day‐time period. This is potentially an effect of increased variability often observed post‐exercise (Francois et al., 2018; Kulawiec et al., 2021; Thomas et al., 2016). Though access to data quantifying the riders' nutritional intake throughout the camp was unavailable, the riders in the present study consumed a meal soon after exercise. Ingestion of carbohydrate‐rich meals inherently raise the concentration of glucose in circulation, a pattern that can be identified in our data in Figure 1. Indeed, in some riders, the ingestion of this meal resulted in transient hyperglycaemia (maximum [iG] data displayed in Table 2). The post‐prandial insulin response instigates a subsequent fall in [iG]. Exercise has been shown to increase glucose uptake through insulin‐independent mechanisms and via increased insulin sensitivity for a number of hours post‐exercise (Borghouts & Keizer, 1999; Kjaer et al., 1984; Maarbjerg et al., 2011; Mikines et al., 1988). The decline in [iG] in this study appears to continue into the night‐time hours until the early hours of the following morning. Fittingly, mean [iG] was significantly lower during the night‐time period when compared against the designated day‐time period. There was also a trend toward larger amounts of TBR (<70 mg/dl) during the night‐time hours, although it did not reach statistical significance.

A number of studies have shown a tendency for lower [BG] during the night‐time hours, perhaps as a reflection of a reduction in sympathetic activity and counter‐regulatory hormone responses (Graveling & Frier, 2017; Iscoe et al., 2008; Jones et al., 1998; Merl et al., 2004). It has also been suggested that the threshold for counter‐regulation of [BG] is lower during sleep (Gais et al., 2003). While this information is of clinical importance regarding people living with metabolic dysregulation, for example, diabetes where nocturnal hypoglycaemia is a common and concerning issue, the health and/or performance implications for athletic populations is unknown. Hence, caution in interpretation is clear given the lack of population‐specific glycaemic ranges.

In this study, some hyperglycaemia was experienced and all TAR occurred during day‐time hours. Mean TAR in our data during the overall 24 h period was 3 ± 1%. Shah et al. (2019) reported a similar proportion of TAR in healthy individuals at 2.1% and Berry et al. (2023) observed even less with 0.3% TAR. At present, there is no established recommendation for TAR in a healthy population. The threshold for TAR in adults with type 1 diabetes is >180 mg/dl and consensus guidelines advise that less than 25% of total daily time should be spent exceeding target range (i.e., >140 mg/dl) (Battelino et al., 2019) Previous work has set a threshold of ≥140 mg/dl to identify groups not currently diagnosed with diabetes but at a heightened risk of developing health complications (The Expert Committee on the Diagnosis and Classification of Diabetes Mellitus, 1997) These thresholds are based on risk factors for potential pathologies but not general health. In young and healthy individuals, it is unlikely that these thresholds would be markedly breached for a substantial time. Therefore, there is debate as to what is the upper threshold for optimal health, particularly in highly athletic individuals.

Worth noting was the proportionate amount of time the riders in this study spent in hypoglycaemia. The athletes displayed an average of 8 ± 2% of time below target range on a daily basis. This is twice that recommended by the international consensus guidelines of 4% total per day (Battelino et al., 2019). As the CGM used in this study has an effective measurement floor of 55 mg/dl, it was unable to quantify any time spent in severe hypoglycaemia (<54 mg/dl). This study was observational and retrospective and no reactive interventional measurements, such as finger stick sampling, were employed to validate sensor concentrations. Hence, it is possible that some of our female riders may have experienced time within this range without us being able to quantify it. With the caveat of ambiguity in a clear definition for hypoglycaemia in those without diabetes, the implications of hypoglycaemia, when termed as <70 mg/dl, on general health and wellbeing outside of a sporting context are well‐documented (American Diabetes Association, 2021; Cox et al., 1993; Graveling & Frier, 2009; Owens et al., 1998). Yet, transference of these findings to highly athletic and professional sports people from both an exercise performance and recovery perspective is missing. Considering the demands of multi‐day activity and the carbohydrate requirements for adequate glycogen replenishment (Burke et al., 2001; Canada, 2000; Coyle, 2012; Jeukendrup, 2014), the occurrence of hypoglycaemia identified by CGM may offer some warning of inadequate carbohydrate intake in the post‐exercise period.

Some of the hypoglycaemic events observed in this study were abrupt, severe and somewhat unexpected. Not only did they fall below the physiological range for sustained periods of time, but their recovery to euglycemia appeared to be quite sudden. The events in question occurred during night‐time hours and due to their unusual pattern warranted some further consideration. Sensors have also been shown to have poorer accuracy when concentrations fall to hypoglycaemic levels. Work by Moser (Moser, Eckstein, McCarthy, et al., 2019) detected a mean absolute relative difference (MARD) of 31.6% during hypoglycaemia in comparison to 16% during euglycaemia. ‘Compression lows’ have also been documented as a potential sensor limitation (Helton et al., 2011; Mensh et al., 2013). As a result, sleeping position cannot be ruled out as a cause for some of the measured TBR during the night‐time period. Therefore, external factors, such as sensor compression from body position or clothing (Röder et al., 2016), skin temperature (Coates et al., 2023) and/or sensor location, must be considered when assessing the possible mechanisms underlying some of the changes that are captured using CGM.

We found no relationship between glycaemic parameters and exercise performance outcomes. Nor were there any associations between glycaemic parameters obtained throughout the night‐time period that preceded a day of training and the next‐day's exercise outcomes. The lack of association between [iG] and exercise metrics is an agreement with Kinrade and Galloway (2021) who observed no association between [iG] and race distance during competition. An important caveat is that the session goal and/or prescribed intensity may have undermined any definitive association between glycaemia and performance outcomes. Studies have shown CGM accuracy to worsen during exercise (Bauhaus et al., 2023; Da Prato et al., 2022; Fabra et al., 2021; Moser, Eckstein, Mueller, et al., 2019) much of the discrepancy between iG and BG measurements can be explained by a lag in sensing (Moser et al., 2017, 2020). This lag may be heightened due to a number of factors many relating to the rapid changes, which occur in the body during exercise. It is possible that such discrepancies may have prevented the identification of any association to exercise performance should they have existed. MARD values of up to 29.8% during exercise have been detected in past studies (Moser, Eckstein, McCarthy, et al., 2019). Hence, further work investigating such relationships under race conditions may provide a better scenario in which to study the area.

Overall, the study identified some possibilities for when CGM may offer greater insight into the glycaemic demands of intensified training. The most obvious being an education tool for individuals and athletes to learn more about their own personal physiology. The impact of food types and meal timing can also be identified using the technology and has generated interest by others (Zignoli et al., 2023). This might help guide nutrition strategies during exercise and in the post‐exercise recovery period (Bowler et al., 2022; DuBose et al., 2020; Kinrade & Galloway, 2021; Podlogar & Wallis, 2022). Due to the considerable and continual high energetic demands of being a professional athlete, low energy availability is a concern for both female and male athletes (Bowler et al., 2022; Logue et al., 2020; Saris et al., 1989). Low energy availability has been associated with observed mild hypoglycaemia (Smith et al., 2016; Thomas et al., 2016). Hence, CGM could act as a potential warning system for chronic inadequate carbohydrate intake if mapped against nutritional intake information.

### 4.1 Study strengths, limitations and possible consideration for future research incentives

Strengths of this study are its inclusion of several consecutive days of glycaemic profiling (via CGM) in an all‐female professional cycling team, which has been stratified into distinct phases and mapped against a quantifiable background of exercise training. However, combined with the appreciably, albeit expected, small participant number, the lack of data on dietary intake and the phase of menstruation are admitted limitations. The influence of carbohydrate intake on glycaemia could be a significant source of glycaemic variance observed throughout a typical day. Future research would benefit from taking these factors into consideration when looking to further our understanding of the glycaemic demands of elite level cycling and its possible interactions with variables such as diet, the female reproductive system, stress and recovery.

CGM sensors have known limitations during exercise (Clavel et al., 2022; Fabra et al., 2021). Most of which have been reported on older generation sensors. Few studies have been completed on newer sensors, which manufacturers claim have improved upon accuracy and reduced delays in sensing. Current CGM technologies are shown to be effective for improving clinical outcomes and they are approved for use in glycaemic management during exercise with those with type 1 diabetes (Moser et al., 2020).

## 5 CONCLUSION

This observational study characterised the iG data of professional female cyclists during a 9‐day training camp. Riders maintained a high percentage of time spent in target range, yet displayed some time <70 mg/dl, that is, hypoglycaemia. Average night‐time glucose concentrations were lower than day‐time values. In addition, glycaemic variability appeared to be greater during day‐time than night‐time. The integration of CGM may help inform a more personalised approach to developing strategies that support performance and recovery in elite athletes undertaking regular bouts of intense exercise during training and/or racing blocks.

## AUTHOR CONTRIBUTIONS

Ross Hamilton, Olivia M. McCarthy, Stephen C. Bain and Richard M. Bracken contributed to the conception and design of the study. Ross Hamilton was responsible for the acquisition and statistical treatment of data. All authors were responsible for data interpretation. Ross Hamilton, Olivia M. McCarthy and Richard M. Bracken co‐wrote the original draft of the manuscript. All authors contributed to revising the article. All authors provided final approval of the version to be published.

## CONFLICT OF INTEREST STATEMENT

The authors declare that they have no conflicts of interest. Supersapiens had no part in the production, design or interpretation of data in this research study.
